# Investigation on Bridging Defects in 3D-Printed Polylactic Acid Beams Using Fused Filament Fabrication

**DOI:** 10.3390/polym18020261

**Published:** 2026-01-18

**Authors:** Hao He, Zhi Zhu, Y. X. Zhang, Richard (Chunhui) Yang

**Affiliations:** 1 Centre for Advanced Manufacturing Technology, School of Engineering, Faculty of Engineering, Computing and Science, Western Sydney University, Locked Bag 1797, Penrith, NSW 2751, Australia; 19208002@student.westernsydney.edu.au (H.H.); 18794915@student.westernsydney.edu.au (Z.Z.); sarah.zhang@uts.edu.au (Y.X.Z.); 2 School of Mechanical and Mechatronic Engineering, The University of Technology Sydney, 81 Broadway, Ultimo, NSW 2007, Australia

**Keywords:** Fused Filament Fabrication (FFF), Polylactic Acid (PLA), bridging defect, buckling, experimental study, analytical modelling, defect detection, evaluation and mitigation

## Abstract

The bridging defects compromise the structural integrity and strength of 3D-printed polymer parts with the Fused Filament Fabrication (FFF) process. Conventional approaches to avoid bridging defects include simply minimising bridging span and/or adding support structures, which greatly limit the freedom and flexibility of designing FFF-printed polymer products. To lift this limit, this study develops a systematic analytical–experimental framework to investigate the formation and evolution of bridging defects in Polylactic Acid (PLA) bridging beam structures printed using FFF and proposes mitigation methods by adjusting FFF print settings and optimising the beam structures’ geometries. The developed analytical models can capture temperature and elastic modulus evolution, as well as strand curvature, where the modelling results show good agreement with experimental measurements, with coefficients of determination, $R^{2}$, of up to 0.9433. Buckling behaviours are also modelled and quantified in terms of girder width, which increases from 1.2 mm to 4.3 mm as the span length increases from 60 mm to 140 mm, respectively. The obtained results indicate that thermally induced residual stress plays a dominant role in triggering structural instability in support-free beam structures, where the gravitational contribution was found to be comparatively small. Key FFF printing factors influencing bridging defects are also identified for practical guidance of defect mitigation.

## 1. Introduction

Polymer-based Fused Filament Fabrication (FFF) is advantageous due to its cost-effectiveness in feedstock, recyclability, and extreme versatility. The technology is not only accepted by industrials but also by hobbyists [1,2]. The fabrication mechanism can be extended to other materials with similar characteristics, like metals, concrete [3], and ceramics [4]. FFF has a larger market potential due to its lower site and operational requirements compared to other 3D printing technologies, such as Stereolithography (SLA), Selective Laser Melting (SLM), and Laser Powder Bed Fusion (L-PBF). However, the defect occurrences during the FFF printing process are impacting its further growth in the market. Research works focused on defects, including dimensional accuracy, delamination, warpages, and bridgings during FFF processes, were conducted, achieving significant progress in detection, reduction, and mitigation. Common defect mitigation involves establishing machine condition monitoring (MCM) systems to prevent failure when those abnormalities are detected. A comprehensive literature review was conducted by the authors in the previous work [5]. To root out these defects, it is essential to understand the mechanisms underlying their occurrence and identify the factors that influence their formation and evolution to mitigate them by adjusting the corresponding factors.

Bridging defects normally occur when the extruded filament spans across two fixed ends without any support material [6]. Sagging is the prominent phenomenon associated with bridging, which is commonly considered the filament droop due to gravity. The filament deformed and curved rather than the designed straight line. The sagging from the deformation not only affects the aesthetics and dimensional accuracy of the 3D-printed part but also reduces the strength of the structure. Moreover, it weakens the adhesion between filaments, leading to delamination and layer separation as a chain reaction. Specifically, the effect of gravity is a dominant deformation mechanism in large-format and high-flow extrusion-based additive manufacturing. Pricci (2025) [7] modelled gravity-induced sagging in support-free bridging using a rheology-based finite element method (FEM) framework implemented in COMSOL for pellet-based extrusion additive manufacturing.

The Design for Additive Manufacturing (DfAM) standards, such as ISO/ASTM 52910 [8], provide general geometry design guidelines, but there are no explicit design limits for the maximum bridge span lengths in the polymer FFF process due to the complexity of filament and printing machine variation. Empirical industry data [9,10] suggests a practical limit of 10 mm for the span length. Also, empirical manufacturing criteria, such as the longest printable bridge length, have been introduced to help in printing path strategy and support generation [11]. The short span significantly constrains the design freedom. The impacts of bridging defects are amplified in the large-flow material extrusion process due to the increased mass and the state of fusion [12]. Compared to other defects, there is less focus on the bridging defects, as designers generally tend to avoid support-free components at the design stage. However, addressing and solving these defects is unavoidable for improving the printing performance and design freedom at a detailed level. This will affect the dimensional accuracy of support-free components, which is especially noticeable in large-scale printing results. Apart from restraining span distance as mentioned in the design guide, a common solution for bridging defects in 3D polymers is applying the support function in the printing. This is designed to be easily disassembled from the 3D-printed part, but the support poses an aesthetic issue, as support components can leave marks on the surface, which requires further post-processing. This can also degrade the dimensional accuracy of the 3D-printed part. Furthermore, it is impossible to cover all bridging or overhang areas using the support, especially at areas within the structure that is generally avoidable for complex geometries. Therefore, inaccuracies or failures in printing internal bridging structures can directly impact the competitiveness of 3D printing.

Another method for alleviating the residue of support on the 3D-printed objects is using dissolvable materials, like polyvinyl alcohol (PVA) [13] and UltemTM 9085 [14], for the support. Ice [15] can also be used as a support, as it melts gradually at ambient temperature. Despite the dissolvable supports can be used to reduce the bridging effect whilst maintaining the surface finish quality, their drawbacks are also evident, such as the cost of customisation and reduced mechanical properties. The compulsory post-processing of dissolving the support structure is also time-consuming. In summary, this method is not effective enough in reducing the cost and simplifying the complexity of the printing process. Regardless of the circumstance, the use of support structures inherently demands additional material, which consequently results in material wastage. Wall et al. (2023) [16] proposed a technical solution by adding ad hoc objects as support to reduce the print time and save material. The technique requires continuous human monitoring of the printing process to add support at the appropriate time. In addition, the dimension precision of the ad hoc material still limits the applicability and convenience of the technique.

Researchers also seek approaches to avoid support structures while maintaining the integrity of the original CAD geometry. For example, the 3D-printed object can be partitioned into printable segments without support [17,18]. However, the bonding process required to adhere to these separated pieces may compromise the structural strength of the final 3D-printed object. Modifying the infill pattern to enhance self-supporting capability can also be considered as a potential method to avoid using external support structures [19]. However, this approach may not be applicable to all geometries. It is especially challenging for bridging structures, which typically feature near-90° overhangs and long spans without underlying support. Changing the printing mechanism may provide a solution to avoid the need for a support structure; for instance, multi-axis 3D printing technology with a dedicated slicer requires no support during printing [20], which could be an accessible approach to reduce bridging defects with such technology. However, to mitigate those bridging defects, workable, feasible, and innovative solutions are still needed, which can be easily implemented on existing FFF printers in the market. Therefore, it is very crucial to identify the root causes of the bridging defects and address the key factors influencing their severity. Experimental studies [21,22,23] revealed that key factors (such as print temperature, print speed, and fan speed) influence bridging defects. Rapid cooling [24], for instance, can mitigate sagging by solidifying and shrinking the extruded filament in air to resist gravitation. Residual stresses due to rapid thermal fluctuations also cause the distortions of the FFF-printed parts [25,26]. The analytical study can be utilised to model the nozzle extrusion process [27,28], which is affected by the nozzle temperature and velocity [27]. Up to now, most of the research reported in the literature was experiment-based, and the analytical models, which can describe the effects of those key factors on the formation and evolution of defects, still remained blank. Additionally, analytical models can help predict the extent of defects, thereby confirming whether the design meets expectations and requirements, ultimately reducing design costs. These models can also help identify the significance of those key factors and seek the optimal combination of parameters. While few comprehensive analytical models were available specifically for bridging defects, prior research established foundational work to address this gap. In this study, analytical models are employed as theoretical analysis tools to characterise and interpret bridging defects by integrating experimental observations. A key challenge in analytically modelling bridging defects lies in their dynamic nature. The material status and loading conditions of structure vary during the printing process. This temporal variability complicates the development of a universal model that is applicable to all potential printing scenarios. To address this complexity, a segmented analytical approach is adopted in this study. Wu et al. (2023) [12] established the Quantitative Fusing Segment (QFS) model and categorised it with two printing statuses based on whether the filament is still in contact with the nozzle (QFS-A) or not (QFS-B). It facilitates the understanding of the loading conditions of the filament in the printing process, but there are still gaps in quantifying each load for in-depth analytical modelling. Machine Learning algorithms offer a robust foundation for analytical modelling by providing an accurate prediction of bridging deformation. Jiang et al. (2019) [22] developed a Back-Propagation Neural Network (BPNN) to predict the bridging deformation under different print speeds, print temperatures, and cooling fan speeds. Ao et al. (2024) [21] applied a Computational Fluid Dynamics (CFD) simulation to predict the sagging distance under given conditions. In the buckling aspect, Torre et al. (2021) [29] developed analytical and experimental works specific to the buckling of FFF-printed PLA parts.

To address the research gaps identified above, this study aims to establish a hybrid analytical–experimental framework to systematically identify the root causes of bridging defects and provide multiple analytical models to analyse the formation and evolution of those defects in FFF-printed PLA. The bridging defects are captured with imaging and digital data, and then using the beam theory, three analytical models are developed to describe the local printing process and the global structural behaviours, respectively. The developed analytical models are further validated and calibrated to enhance their practicality with experimental data. While gravity is commonly acknowledged as the main cause of the sagging effect, buckling is also identified as a crucial factor for the bridging deformation. Wolfs and Suiker (2019) [30] studied buckling during the concrete 3D printing process and predicted the length of critical buckling under simply supported thin-wall and free-wall boundary conditions with excellent agreement between the FEM simulation and experimental values. As observed, inadequate structural strength combined with residual thermal stress can trigger buckling, which aggravates the bridging deformation. The mutual interaction between buckling and other bridging defects makes it challenging to derive an explicit analytical solution for this system. However, the warping studies can help develop an effective solution, as the warping defect’s formation is based on a similar mechanism of bridging defects. The bending moment is another cause of the warping defect [31]. Although the bending moment is generated in different ways, gravity mainly causes a bending moment that further leads to bridging, and residual stress mainly causes warping. Some parametric studies showed several practical warping mitigation approaches that increase print speed [26] and layer thickness [26,31,32] to be constructive in bridging defects. Other factors, such as build plate temperature, chamber temperature, and nozzle temperature, also play significant roles in warping formation. This can be mitigated by finding the optimal print settings [33,34]. In this study, the critical conditions of buckling horizontally are identified, and the results also provide the potential to refine the common rules of thumb for Design for Additive Manufacturing (DfAM). This paper is structured with four main sections. Section 2 details the methodology developed in this study to investigate the causes and formations of bridging defects. Section 3 shows temperature evolutions of the local printing process and the derived evolutions of the elastic modulus according to the temperature-dependent characteristics. Conclusions and recommendations for future work are provided in Section 4.

## 2. Methods

**Figure 1:**
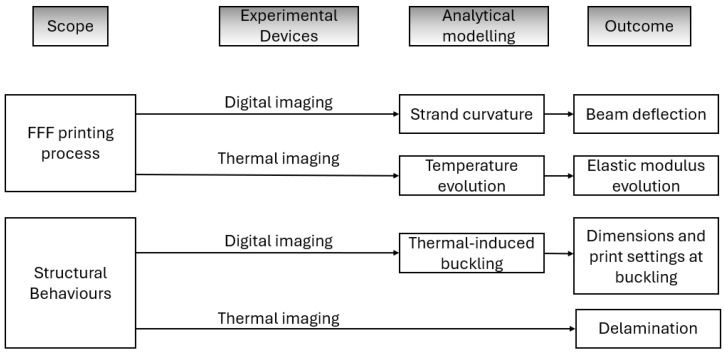
Schematic of the devised hybrid experimental–analytical framework.

This study develops a systematic framework combining experimental studies and analytical modelling to investigate root causes of the bridging defects in FFF-printed PLA and provide solutions to minimise them. The schematic of this approach can be found in Figure 1. The study focuses on both the FFF printing process and the FFF-printed PLA bridging parts to investigate their deformations during the process and the formation and evolution of the defects on them. The data for the study is collected with two digital imaging systems: digital imaging and thermal imaging. Two types of events are recorded: the local event is for the printing process on the single strand, whereas the global event treats each strand as an entity. Based on the recorded images, three analytical models are developed to describe the strand curvature, the temperature evolution, and critical buckling load conditions, respectively.

The outcomes of this hybrid experimental–analytical analysis framework of FFF-printed PLA bridging beams include the beam deflection, temperature-dependent elastic modulus evolutions, geometric dimensions, print settings, and defects. These extracted results help identify the key factors and parameters, including both material and/or structure and the FFF printing process of FFF-printed PLA parts. They can be used to optimise the FFF printing process by adjusting the settings of the key printing parameters and altering the design features of the parts, considering the principles of Design for Additive Manufacturing (DfAM).

### 2.1. Experimental Setup

#### 2.1.1. Selection of Filament Material, 3D Printer, and Measurement System

**Figure 2:**
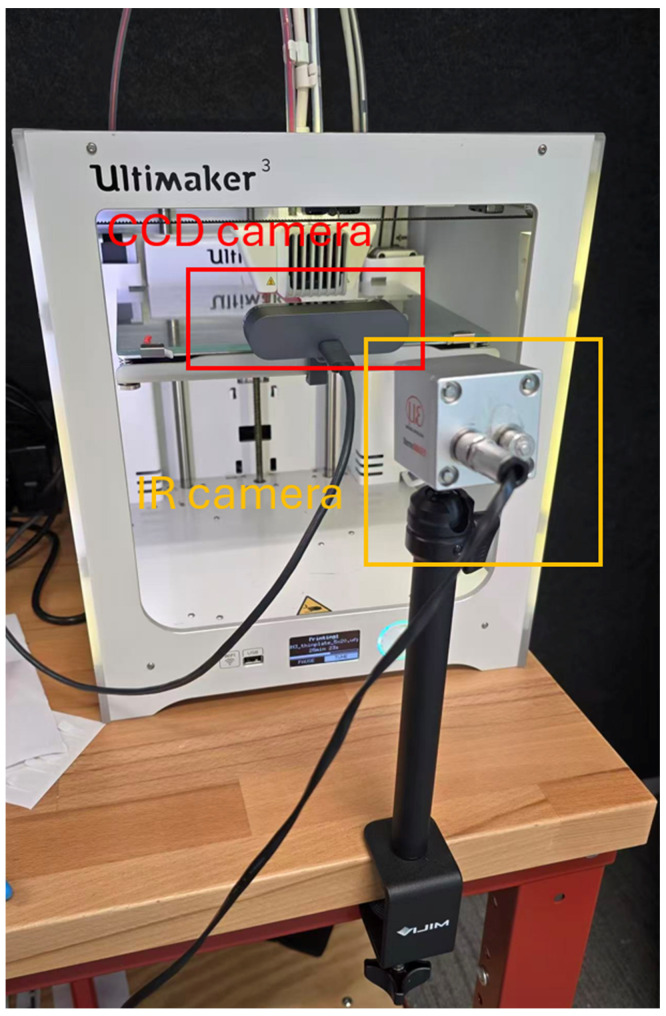
Printing process image device setup.

The commercial TECOR PLA+ 3D filament (2.85 mm, grey and red) from InkStation, Bevery Hills, NSW, Australia was selected as the featured filament material, and for digital imaging, two commercial FFF 3D printers were used for printing test samples in this study. The FFF printing processes of the test samples were recorded with digital and IR cameras, and their settings are shown in Figure 2.

Table 1 summarises the key printing parameters used in this study. The parameters were maintained at the default settings recommended by the printers, and they were modified and specified under the necessary circumstances accordingly. This approach ensured stable extrusion, process repeatability, and consistency across various testing cases.

The Logitech^®^ (Lausanne, Switzerland) BRIO 4K USB camera was mounted to the glass build plate. It was operated at a resolution of 640 × 480 pixels to balance data quality and processing time efficiency. Both images and recordings were captured to record the formation and evolution of deformations and defects in the FFF-printed bridging beams, and they were later used to validate the devised analytical models. The IR camera Micro-Epsilon^®^ (Ortenburg, Germany) TM160 with a resolution of 160 × 120 pixels, a minimum measurement distance of 0.2 m, and a lens with a 5 mm focal length was installed to record temperature distributions over time for the printing test samples. It is worth pointing out that the IR camera was primarily used to capture the relative temperature distribution and cooling behaviour rather than to determine absolute temperature values; therefore, the selected instruments remain suitable for analysing thermal evolution trends.

#### 2.1.2. Design and FFF Printing of Test Samples

**Figure 3:**
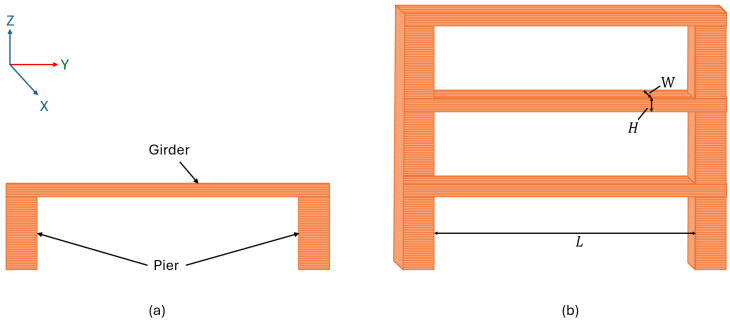
(**a**) Bridging defect test beam sample and (**b**) stacked test sample.

Two single-girder and multi-girder bridging beam structures were designed to study the bridging defects via monitoring and recording their printing processes, as shown in Figure 3a,b, respectively. The test artefact design was based on the authors’ previous work [35]. In these test samples, the piers in the bridging beam structure serve as the primary supports of the entire test sample structure. The dimensions of the girder component (length *L*, width *W*, height *H*) are treated as the primary variables to examine their influence on deformation behaviour under support-free bridging conditions. To examine the deformations when the bridging defects were generated along the girder component spans between two piers, these samples were designed to be printed without additional supports in the bridging beams. Multi-girder bridging beam test samples, as depicted in Figure 3b, are employed to further investigate bridging defects by systematically varying the girder width (*W*) and height (*H*). Compared with the single-girder configuration, the multi-girder design enables the investigation of inter-girder interaction, adhesion, and collective deformation behaviour, which are essential for capturing buckling and delamination phenomena under support-free bridging conditions.

As for FFF printing of the test samples, the infills were set as the line pattern, and the raster angle was set as 90° to ensure that the girder components were printed in straight lines. The dimensions of the girders were varied to seek their influences on the deformations when bridging defects were generated and evolved. The girder component was designed as a cuboid with three key geometric dimensions, length *L*, width *W*, and height *H*, respectively, as detailed in Table 2. The span lengths *L* were chosen to cover three unsupported bridging conditions commonly encountered—short, medium, and long in the practical FFF printing, and the length remained in the stable printing range of the selected FFF printers. The width *W* and height *H* values were defined over a broad range to capture local and global deformation behaviours. The range includes buck-scale dimension (25 mm), as well as a single extrusion thickness (0.2 mm). The small dimension range (0.2–1 mm) was intentionally concentrated as it was considered the most sensitive range for structural instability behaviours to occur. Discrete dimension increments were adopted to ensure consistent slicing and deposition tracks, and the line pattern was also applied to the girder component to mitigate irregular strand spacing. These parametric designs and print settings enabled clearer identification of various defects, including bridging, buckling, and delamination.

### 2.2. Investigation of Bridging Defects’ Formations and Evolutions

#### 2.2.1. Curvature Evolution of Single Strand

**Figure 4:**
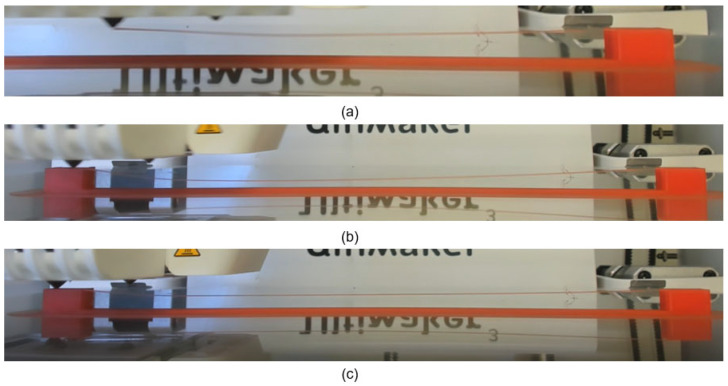
The curvature evolution of the FFF-printed single strand while (**a**) printing on air, (**b**) just arriving at the other pier, and (**c**) after printing.

The FFF printing process of the single strand was captured and analysed to evaluate its deformation characteristics (see Figure 4). It was observed that the extruded material exhibited initial drooping immediately after extrusion from the nozzle, as shown in Figure 4a. As the nozzle moved toward the other pier of its trajectory, the droop reached its maximum extent, as shown in Figure 4b. After the nozzle arrived at the other pier and the deposition was complete, a partial reduction of deflection was observed, as shown in Figure 4c. Such a reduction can be attributed to tension-induced filament realignment between the two fixed ends, where the material remains in a softened state. These research findings provide insights into the formed curvature of a single strand and highlight the influence of material properties and FFF printing process parameters on its final geometry shape.

**Figure 5:**
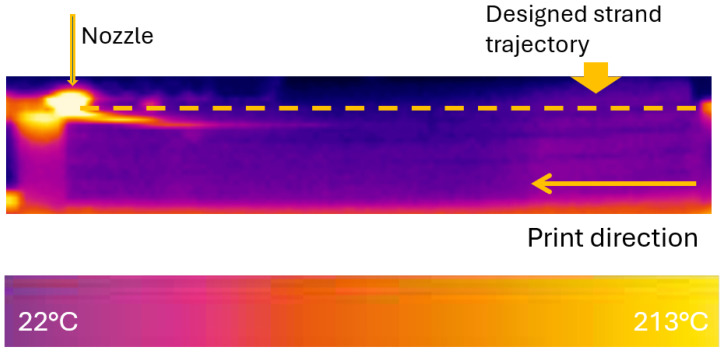
Single-strand temperature distribution when the nozzle finishes printing.

Figure 5 illustrates temperature distributions in the FFF printing field when a single strand was printed across the piers. The strand cools rapidly to ambient temperature since it does not directly contact the build plate, as most printing parts do.

#### 2.2.2. Structural Responses of Multiple Strands and Bridging Beam Structures

The deformations of multiple strands developed in a significantly different way compared to the single strand, and this indicates that gravitational forces and material melting may not be the primary contributors to deformation under these conditions. Several potential causes of deformation development were revealed in the FFF printing process of the multiple strands. Inter-strand adhesion plays a crucial role in maintaining structural integrity, mainly depending on nozzle temperature and print speed [36], which is assessed by visual-based qualitative clarification. Specifically, the outcomes are identified as two types: minor defects, where the FFF-printed structure remains intact but exhibits visible deformation, and major failures, with severe delamination and damage to structural stability.

**Figure 6:**
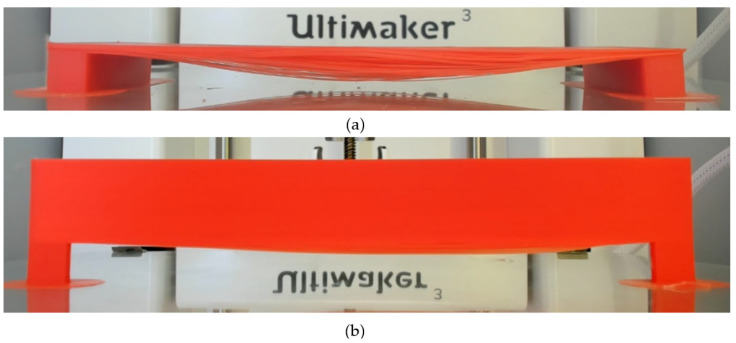
Bridging conditions: (**a**) major (failure) and (**b**) minor (defect) on test samples.

As shown in Figure 6, two types of bridging defects were defined in this study: (a) major failure corresponds to a severe case with distinct deformation, clear delamination, and structural collapse, and (b) a minor defect indicates a case where the bridging structure remains continuous and load-carrying, with limited curvature but no visible inter-strand separation. 

The excessive failures of adhesion cause the failure of the entire structure, as shown in Figure 6a, and clear delamination between the FFF-printed multiple strands was observed. The deflection becomes worse compared to those results with minor adhesion conditions, as shown in Figure 6b. The observed delamination leads to a noticeable reduction in the structural integrity of the pier. It is noted that the bridging condition is assessed qualitatively based on deflection, slope, and delamination defect, but no quantitative mechanical testing was performed on the FFF-printed parts in this study.

Natural sagging makes the adhesion between filaments challenging in the support-free printing process. While the just extruded filament has the potential to adhere to the previous layer and mitigate the deflection, the risk of failure to adhere increases with the accumulation of the FFF-printed strands. During the FFF printing process of the stacked test samples, it can be noted that one pier was dragged and deformed toward the other one. Those unwanted deformations that happened in the piers were a result of the dynamically increasing weight and bending moment generated by the multiple strands of the girder. Note that the weight of the strands is much lower and thus ignored compared to the generated bending moments. Additionally, the bending moments also aggravated the warpages at the base component.

**Figure 7:**
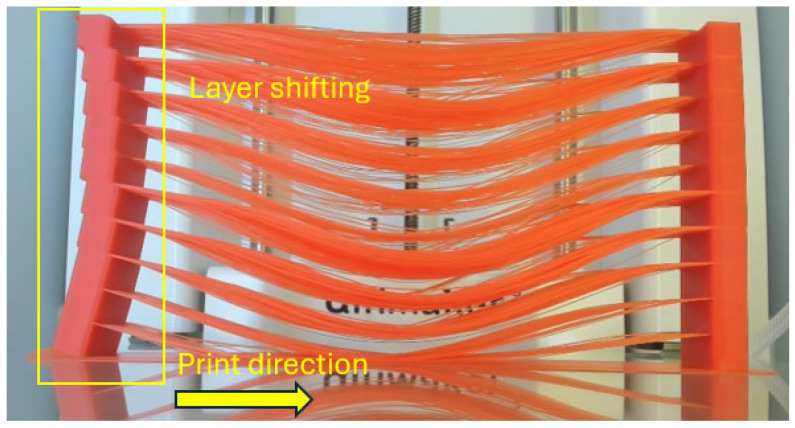
Failed stacked test sample with evident layer shifting and buckling.

As shown in Figure 7, layer shifting was observed in the stacked test samples, particularly in the layers containing girder components. This phenomenon could be attributed to accumulated tensile stresses induced by the strands’ weight and temperature changes. The observed layer shifting demonstrates that such progressively increasing stresses can critically impair the structural integrity of the FFF-printed parts. In addition to the layer shifting, bending-induced deformations in pier components were also clearly observed. Although the brim support at the beginning layers was added to stabilise the test samples, it was insufficient to counter the progressive mechanical loads during printing, ultimately leading to the warping of the brim and subsequent bending of the piers.

**Figure 8:**
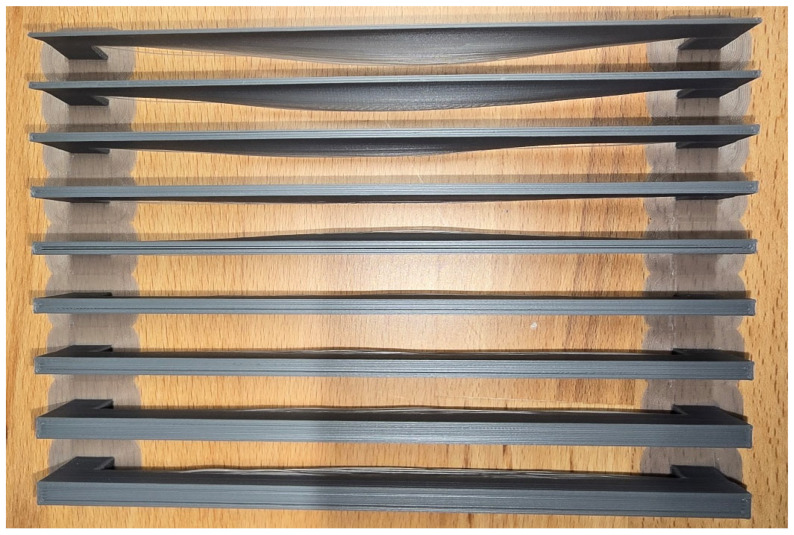
Horizontal buckling of the girder component.

When the adhesion between the pier and the build plate is too weak to maintain structural stability, the pier is tilted inward toward the girder component, reducing the span and further compressing the girder component. Buckling occurs when compression exceeds the critical condition. It can develop in both transverse and horizontal directions, as shown in Figure 8, which depends on the aspect ratio of the rectangular cross-section shape. The deformation tends to occur along the smaller aspect ratio of width and height, which is consistent with the typical buckling behaviour. The continuous curvature of the buckling also indicates that the deformation occurs gradually, induced by the progressive release of thermal residual stresses.

**Figure 9:**
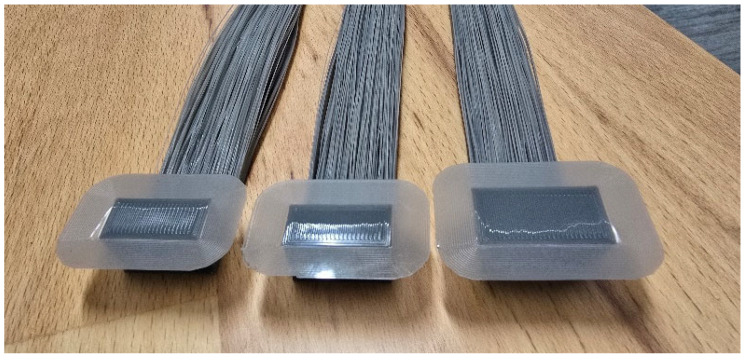
Warpage on the pier component.

Buckling deformation can be aggravated by inadequate adhesion between the pier component and the build plate, as shown in Figure 9. The directional pattern of the warpage provides direct evidence of buckling-induced deformation, showing inward displacements of the pier component toward the girder.

Several scenarios were recorded as valuable for thermal analytical modelling and analysis. The following thermal images were captured with the same reference temperature range (30–60 °C) for comparison. The following cases are highlighted for discussion due to their distinctive characteristics.

**Figure 10:**
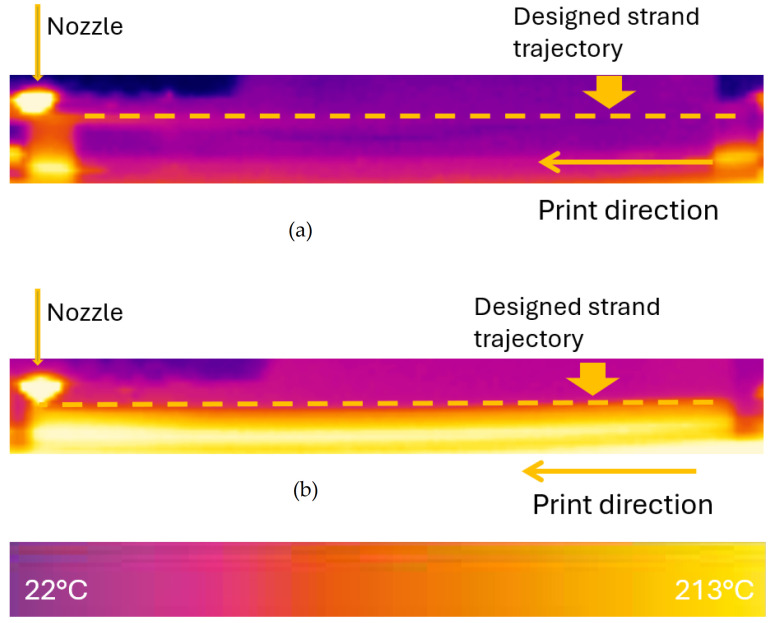
Thermal distributions of (**a**) bridging test sample and (**b**) control sample with the same testing dimension, respectively (all printing parameters are kept identical for both conditions, unless otherwise stated).

To understand the thermal effects on the initiation and evolution of the bridging defects, a control sample with the same dimensions as the girder component was printed directly on the printing bed. It was observed that the average temperature of the girder component was significantly lower than that of the control sample. The temperature of the girder component closely approached the ambient temperature, as shown in Figure 10a, whereas the control sample was maintained at a temperature close to the printing bed temperature shown in Figure 10b. For both conditions, identical printing settings were applied, as provided in Table 1. This observation confirms that the girder component cooled rapidly below the glass transition temperature of PLA. While rapid cooling effectively mitigates sagging in bridging structures, it simultaneously impairs interlayer adhesion due to reduced polymer interdiffusion. The rapid cooling cycles on the printing part generate non-uniform temperature gradients. These gradients induce residual stresses between layers and weaken the strands’ adhesion, ultimately leading to interlayer fracture and delamination [26].

### 2.3. Analytical Modelling

Analytical modelling in this study focuses on the short-time thermo-mechanical behaviour occurring immediately after strand deposition. The modelling framework is established to capture the transient cooling, rapid stiffness evolution, and deformation response within a very short time scale, rather than long-term viscoelastic or creep-dominated behaviour. Consistent with the use of small-time increments in the thermal analysis and the experimentally observed rapid early-stage cooling, the subsequent deflection and buckling models are formulated to describe the post-deposition stage, where the bridge span is fully developed and the dominant deformation governs the final geometric integrity of the printed structure.

#### 2.3.1. FFF Printing Process

*Non-linear filament thermal behaviours (heat distribution) during the FFF printing*

Understanding the thermal behaviour of FFF-printed PLA is the starting point to establish the analytical models, where the study of the single strand is the foundation of the thermal study of complex multiple-strand cases. The thermal behaviour is mainly affected by environmental temperature, including both chamber temperature and build plate temperature, as well as nozzle temperature, which can be considered the initial temperature of the extruder. Comprehensive analysis of the temperature evolution of a single strand extruded on a build plate was performed by experimental results and analytical modelling [24,37]. The temperature was observed to drop rapidly from the nozzle temperature to gradually reach the ambient temperature. The cooling rate was found to be much lower when the extrusion rate increases [38].

This temperature evolution-based analytical model is useful when the strand has no direct contact with the heating surface. The printing strand of the girder components dissipates heat primarily via convection and radiation. The governing equation of the heat transfer rate [39] can be represented as follows:

(1) $$\frac{\partial Q}{\partial t}={\dot{Q}}_{cond}+{\dot{Q}}_{conv}+{\dot{Q}}_{rad}$$

where ${\dot{Q}}_{cond}$ is the conduction rate based on Fourier’s law. For the segments in the single strand, it goes through the whole filament. ${\dot{Q}}_{conv}$ is the thermal convection rate based on Newton’s law of cooling [40], and the radiation rate ${\dot{Q}}_{rad}$ is based on Stefan-Boltzmann’s law of radiation.

This model can be solved with a discrete element analysis method because some material properties cannot be simply represented by multiple numerical properties [41], so the left rule of the Riemann sum was applied. The following model describes the temperature changes ΔT with a very short interval Δt for an arbitrary section n in the printing strand at printing time t. Based on the results of previous research [39] and the analysis, Equation (1) can be integrated as follows:

(2) $${c_{p}}_{T}m_{s}\Delta T={(kA_{sec}(T_{n-1,t}-T_{n+1,t})/L_{s}+h}_{T}{A_{s}}_{T}\left(T_{t}-T_{\infty }\right)+\varepsilon {A_{s}}_{T}\sigma (T_{t}^{4}-T_{\infty }^{4}))\Delta t$$

where ${c_{p}}_{T}$ is the heat capacity of the filament at temperature T, $m_{s}$ is the mass of the strand segment, k is the thermal conductivity of the filament material, $A_{sec}\;$ is the sectional area of the strand segment, $T_{n-1}$ and $T_{n+1}$ are the temperature of the contacted strand segments, $L_{s}$ is the length of the strand segment, $h_{T}$ is the heat transfer coefficient at temperature T, $A_{s}$ is the surface area of the strand segment, $T_{t}$ is the internal filament temperature at time t, $T_{\infty }$ is the ambient temperature, ε is the emissivity or absorptivity, and σ is the Stefan–Boltzmann constant.

Most heat is dissipated by convection in this case, as low conduction is caused by the limited contact surface of the filament. Hence, Equation (2) can be simplified as follows:

(3) $${c_{p}}_{T}m_{s}\Delta T\approx (h_{T}A_{s}\left(T_{t}-T_{\infty }\right)+\varepsilon A_{s}\sigma (T_{t}^{4}-T_{\infty }^{4}))\Delta t$$

Hence, the cooling time increment Δt required to decrease the temperature deviation ΔT at the temperature $T_{t}$ for an arbitrary section can be represented as follows:

(4) $$\Delta t(T_{t})=\frac{{c_{p}}_{T}m_{s}\Delta T}{\left(h_{T}\left(T_{t}-T_{\infty }\right)+\varepsilon \sigma \left(T_{t}^{4}-T_{\infty }^{4}\right)\right)A_{s}}$$

By numerically integrating the time Δt with given initial conditions, the temperature change over time, T(t), can be modelled as follows:

(5) $$T_{n,t}=T_{n,t-\Delta t}+\Delta T\cdot \Delta t\left(T_{t}\right)$$

**Figure 11:**
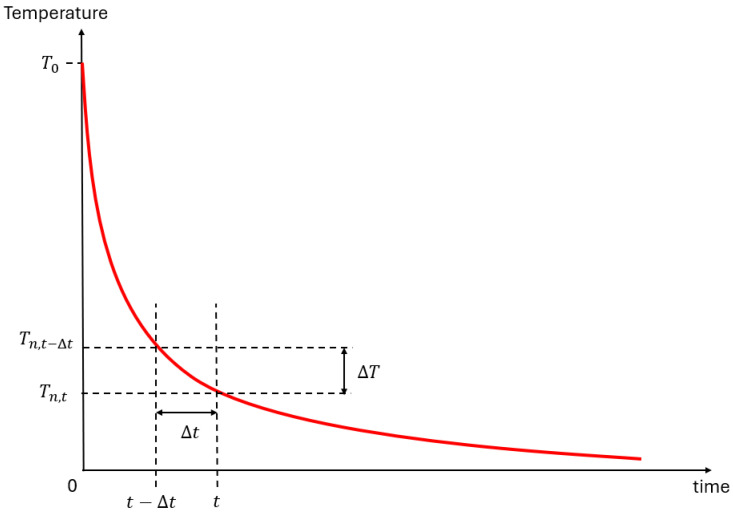
Diagram of the temperature evolution.

There is an initial boundary condition: $T_{n,0}=T_{0}$. Figure 11 illustrates the temperature evolution over time, as dictated by Equation (5).

**Figure 12:**
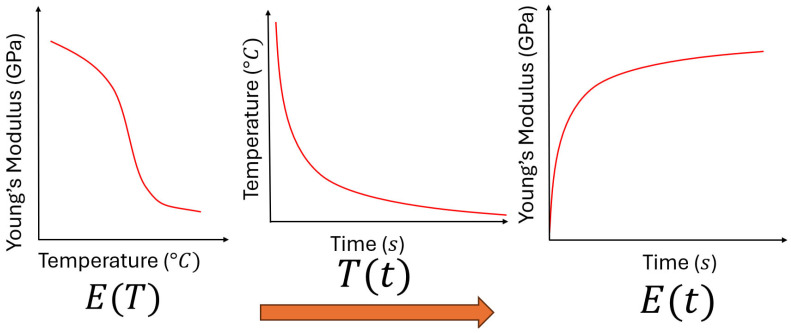
Schematic diagram of deriving E(t) as the function of time by substituting the experimentally measured T(t) into the material property E(T) as the function of temperature.

Similarly, once the temperature-dependent elastic modulus E(T) obtained from temperature-dependent material data is combined with the experimentally measured temperature evolution T(t), the time evolution of elastic modulus E(t) can be derived, as depicted in Figure 12.

The thermal modelling parameters and assumptions adopted in this study are summarised in Table 3:

*Deflection of the bridging beam*

Analysing the pattern of local curvature provides insight into the underlying loading condition. The bridging beam deflection is considered the most appropriate structural behaviour for the case, which can be described using the following differential equation that governs the beam deflection under a uniformly distributed load ω as follows:

(6) $$\frac{d^{4}y}{dx^{4}}=\frac{\omega }{E(t)I}$$

where *ω* is the uniformly distributed line load (N/m), $\omega =\frac{mg}{L_{o}}$, with the single strand mass *m*, gravitational acceleration g, and the length of span $L_{o}$. E(t) is the Elastic modulus of the filament, which is a function of printing time t, and I denotes the moment of inertia for a round cross-section in the beam.

By integrating this equation successively, the third-order, second-order, and first-order differential equations can also be obtained, which describe the shear force V, bending moment M, slope of deflection θ, and deflection of the beam *y*, respectively, as follows:

(7) $$\left\{\begin{array}{c} \begin{array}{c} \frac{V}{EI}=\frac{d^{3}y}{dx^{3}}=\frac{\omega }{EI}x+C_{1}\\ \frac{M}{EI}=\frac{d^{2}y}{dx^{2}}=\frac{\omega }{2EI}x^{2}+C_{1}x+C_{2}\\ \theta =\frac{dy}{dx}=\frac{\omega }{6EI}x^{3}+\frac{C_{1}}{2}x^{2}+C_{2}x+C_{3} \end{array}\\ y=\frac{\omega }{24EI}x^{4}+\frac{C_{1}}{6}x^{3}+\frac{C_{2}}{2}x^{2}+C_{3}x+C_{4} \end{array}\right.$$

where $C_{1},\;C_{2},\;C_{3}$ and $C_{4}$ are constants from the integrations, and they can be determined using boundary conditions. When the nozzle moved to the other pier and the printing was completed, the strand could be considered as a beam deflection fixed on both sides under a uniformly distributed load. The boundary conditions are defined as follows: (a) fixed support on the pier end (x=0), $y\left(0\right)=0$ and $\frac{dy}{dx}\left(0\right)=0$, and (b) fixed support by the other pier (x=L), $y\left(L\right)=0$ and $\frac{dy}{dx}\left(L\right)=0$.

The four constants are then determined as $C_{1}=-\frac{\omega L}{2EI},\;C_{2}=\frac{\omega L^{2}}{12EI},C_{3}=0,C_{4}=0$.

In this case, the deflection curvature can be represented as follows:

(8) $$y\left(x\right)=\frac{\omega }{24EI}x^{4}-\frac{\omega L}{12EI}x^{3}+\frac{\omega L^{2}}{24EI}x^{2}$$

The results from the proposed model will be compared with the experimental data to validate its applicability.

#### 2.3.2. Global Structural Responses

*Buckling*

The force required to trigger the first mode of buckling can be calculated with Euler’s critical stress as follows:

(9) $$\sigma_{cr}(t)=\frac{n\pi^{2}E\left(t\right)I}{{L(t)}^{2}A}$$

where L(t) is the printed filament length and n is the factor linked with the boundary conditions at both ends of the filament during the printing, respectively. There are two end conditions we can have.

- The filament has one end fixed with the column while printing, and the other end has a free flow from the nozzle, and in this case, n = 0.25 should be set.
- After the printing, the completely printed filament has both ends fixed to two columns, and we can take n = 4 for this case.

It is noted that the Euler buckling formulation applied in this study is a first-order estimation of the critical instability condition. Although the printed filament exhibits temperature-dependent and spatially non-homogeneous properties, these effects primarily influence the longer-term transition from a molten to a solidified state. This analysis only serves to investigate the onset of buckling; the model is, therefore, reasonably simplified and treated as an equivalent homogeneous structure over a very short time interval corresponding to the buckling event.

This assumption is considered appropriate for the current investigation, as the buckling behaviour of interest occurs after partial solidification, when the filament has developed sufficient stiffness to sustain compressive loading.

The thermal stress $\sigma_{T}$ can be calculated as follows:

(10) $$\sigma_{T}(t)=\varepsilon E(t)=\alpha \Delta TE(t)$$

where *α* is the coefficient of thermal expansion (CTE).

**Figure 13:**
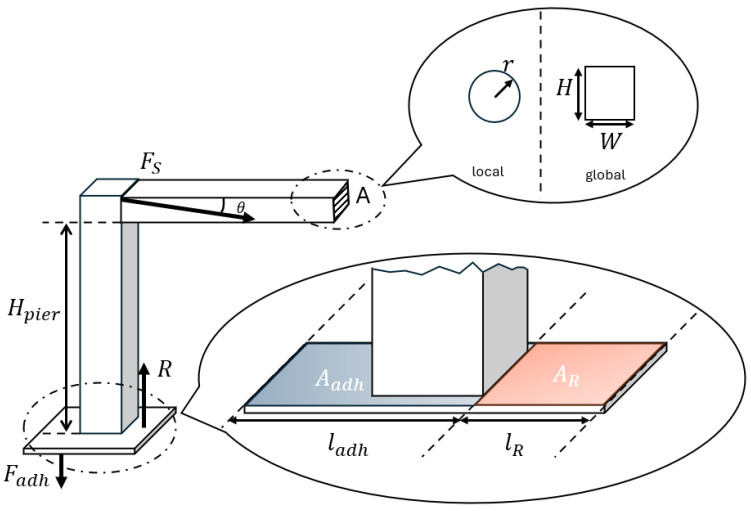
Schematic of force analysis at the buckling critical condition.

The shrinkage of the filament due to the rapid cooling at the nozzle extruder results in tensile thermal stress. Meanwhile, the gravitational effect on the printed filament can be counted through its component along its horizontal orientation. The adjacent pier also provides the structural support to prevent the occurrence of buckling, as depicted in Figure 13.

The moment equilibrium condition can be considered as follows:

(11) $$H_{pier}F_{S}=F_{adh}\frac{l_{adh}}{2}+R\frac{l_{sup}}{2}$$

where $H_{pier}$ is the pier height until the girder. The moment from the base is composed of the adhesion. $F_{adh}$ and the reaction R have an arm that is half the length of $l_{adh}$ and $l_{R}$, respectively. $F_{adh}$ is proportional to the contact area $A_{adh}$ and $l_{adh}$. $F_{S}$ is the summation force from the strands of the girder, which depends on the direction of buckling and is observed as follows:

(12) $$F_{S}=\left\{\begin{array}{c} \sigma_{T}\left(t\right)A+m(t)gsin\theta \;\mathrm{Transverse}\\ \sigma_{T}(t)A\;\mathrm{Horizontal} \end{array}\right.$$

where A is the cross-sectional area of the printed filament and θ is the angle between the curvature and the horizontal line. They can be obtained either from the calculation of the local printing process in the last section or measured from the experiments. The filament mass m of the printing process is also a function of the printing time t, m(t), and it can be linked with its time-dependent length, L(t), which is linear with the time *t* and the printing speed v. They can be described by $m\left(t\right)=\;\rho \cdot L\left(t\right)\cdot A$ and $L\left(t\right)=v\cdot t$.

The reaction force R and interlayer adhesion are dynamic during the printing process and difficult to represent accurately. They may be weakened under warping or inadequate structural adhesion, as shown in Figure 9. To simplify the analytical modelling, the bending moment associated with the pier is written as $M_{p}$ as follows:

(13) $$M_{P}=F_{adh}\frac{l_{adh}}{2}+R\frac{l_{R}}{2}$$

Finally, the general equation of the critical load to trigger the buckling can be determined as follows:

(14) $$F_{cr}=F_{S}-M_{p}/H_{pier}$$

where the amount of force required in the buckling critical condition is $F_{cr}=\sigma_{cr}\left(t\right)A$.

Furthermore, the explicit equation for both transverse and horizontal directions can be derived as follows:

(15) $$\sigma_{cr}(t)A=F_{S}-M_{p}/H_{pier}$$

Therefore, the general equations of the critical conditions to trigger the buckling can be obtained as follows:

(16) $$\left\{\begin{array}{c} \frac{n\pi^{2}E\left(t\right)I}{v^{2}\cdot t^{2}A}=\;\alpha \Delta TE(t)+\;\rho \cdot v\cdot t\cdot gsin\theta -M_{p}/(H_{pier}A)\;\mathrm{Transverse}\\ \frac{n\pi^{2}E\left(t\right)I}{v^{2}\cdot t^{2}A}=\;\alpha \Delta TE(t)-M_{p}/(H_{pier}A)\;\mathrm{Horizontal} \end{array}\right.$$

For the local printing process (n=0.25), the objective is to identify the critical speed that may trigger buckling. The elastic modulus E(t) is modelled with discrete step changes at time intervals Δt to facilitate numerical analysis. The cross-section A can be treated in a round shape with a constant radius, r, for simplicity. The area moment of inertia I and the cross-sectional area *A* can be written as $I=\;\frac{1}{4}\pi r^{4}$ and $A=\;\pi r^{2}$, respectively.

Furthermore, by substituting I and A, the following is obtained:

(17) $$\left\{\begin{array}{c} \frac{\pi^{2}r^{2}E\left(t\right)}{16v^{2}\cdot {\Delta t}^{2}}=\;\alpha \Delta TE(t)+\;\rho \cdot v\cdot \Delta t\cdot g\cdot sin\theta -\frac{4M_{p}}{H_{pier}\pi r^{2}}\;\mathrm{Transverse}\\ \frac{\pi^{2}r^{2}E\left(t\right)}{16v^{2}\cdot {\Delta t}^{2}}=\;\alpha \Delta TE(t)-\frac{4M_{p}}{H_{pier}\pi r^{2}}\;\mathrm{Horizontal} \end{array}\right.$$

It can be found that a higher speed will reduce the critical condition of buckling. At the start of the extrusion, a low elastic modulus due to high temperature causes a high tendency for buckling. Therefore, determining the maximum critical speed is essential to prevent buckling during the printing process. For the printed test artefact (n=4), the buckling condition can also be calculated with variant I and A: $I=\left\{\begin{array}{c} \frac{WH^{3}\;}{12}\;\mathrm{Transverse}\\ \frac{HW^{3}}{12}\;\mathrm{Horizontal} \end{array}\right.$ and A=W·H. 

Where W and H are the width and height of the cross-section of the girder components depicted in Figure 3, and the elastic modulus is stabilised at the ambient temperature as $E_{0}$. Therefore, we can obtain a relation between the dimension and printing speed at the critical conditions as follows:

(18) $$\left\{\begin{array}{c} \frac{\pi^{2}E_{0}H^{2}}{3{v^{2}t}^{2}}+\frac{M_{p}}{H_{pier}WH}=\;\alpha \Delta TE_{0}+\;\rho \cdot v\cdot t\cdot g\cdot sin\theta \;\mathrm{Transverse}\\ \frac{\pi^{2}E_{0}W^{2}}{3{v^{2}t}^{2}}+\frac{M_{p}}{H_{pier}WH}=\;\alpha \Delta TE_{0}\;\mathrm{Horizontal} \end{array}\right.$$

For the FFF-printed samples, the span lengths are fixed; therefore

(19) $$\left\{\begin{array}{c} \frac{\pi^{2}E_{0}H^{2}}{3{L_{0}}^{2}}+\frac{M_{p}}{H_{pier}WH}=\alpha \Delta TE_{0}+\rho \cdot v\cdot t\cdot g\cdot sin\theta \;\mathrm{Transverse}\\ \frac{\pi^{2}E_{0}W^{2}}{3{L_{0}}^{2}}+\frac{M_{p}}{H_{pier}WH}=\alpha \Delta TE_{0}\;\mathrm{Horizontal} \end{array}\right.$$

## 3. Results and Discussion

### 3.1. Temperature and Elastic Modulus Evolution with Time

The time-dependent elastic modulus E(t) was obtained by substituting the experimentally measured temperature evolution T(t) into a temperature-dependent elastic modulus E(T) reported by Zhu et al. (2022) [43].

**Figure 14:**
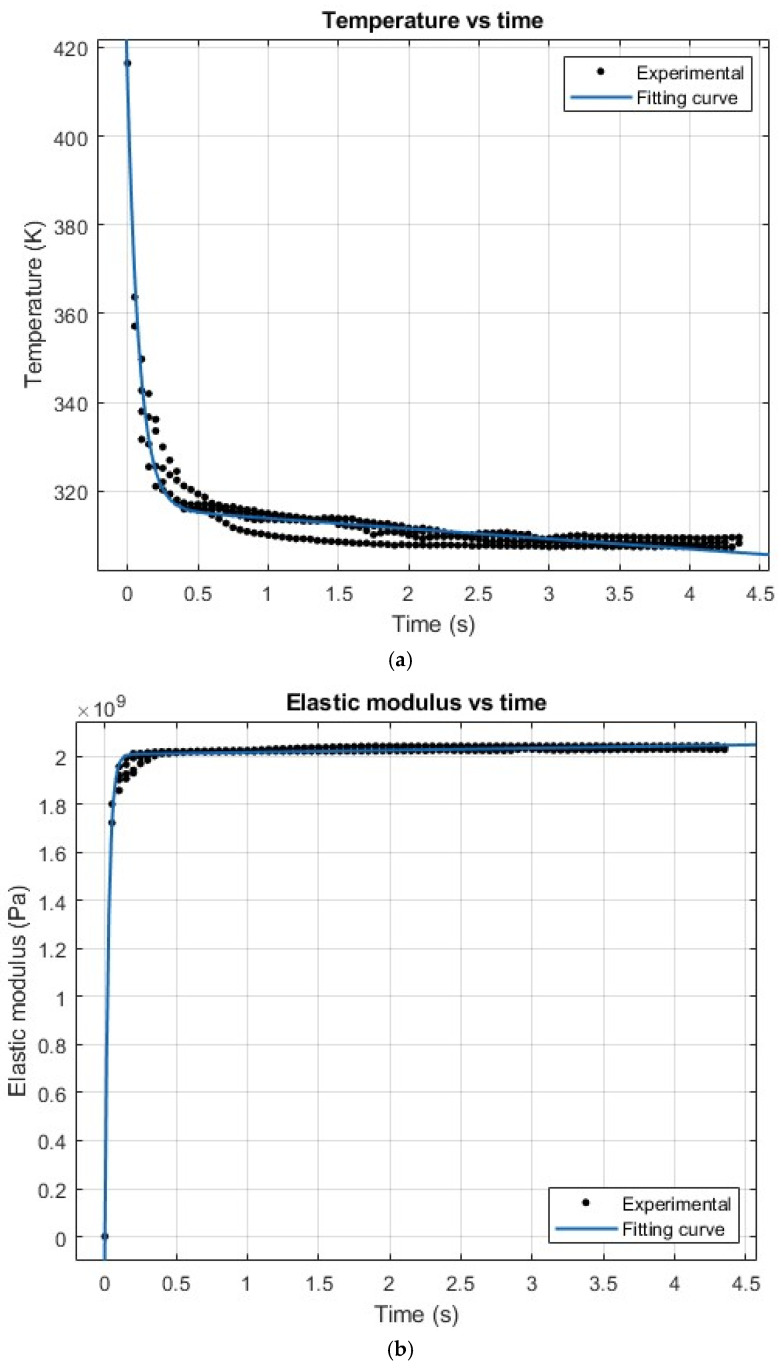
Comparison between experimental data and fitting results: (**a**) temperature evolution and (**b**) elastic modulus evolution over time. Note: the experimental data in (**a**) were obtained from repeated printing trials under identical conditions (three trials), with a bridge span length of 140 mm, and all other printing parameters were kept consistent with those listed in Table 1.

The rapid cooling also means that the majority of the printing process is under the glass transition temperature $T_{g}=60\;{}^{\circ}C$. Hence, the viscoelastic behaviour can be neglected. It can be observed that the curves of time-dependent behaviour of temperature T(t) meet Newton’s law of cooling, and the elastic modulus E(t) follows with a power–law relationship. The curves have been plotted and compared, as depicted in Figure 14.

The temperature evolution T(t) can be fit with an exponential curve, as shown in Equation (20). The cooling behaviour of unsupported PLA strands was found to be dominated by convective heat transfer, which follows Newton’s law of cooling, leading to an exponential decay pattern.

(20) $$T\left(t\right)=ae^{bt}+c$$

The elastic modulus evolution E(t) was represented by a power–law relationship to empirically capture the non-linear strength change associated with temperature-dependent solidification.

(21) $$E\left(t\right)=at^{b}+c$$

where a, b, and c are the constants of the exponential curves, respectively. The fitting curve values are shown in Table 4.

The experimental result is compared to the analytical modelling. From the comparison of the results, it can be observed that the experimental results have a higher cooling rate than the analytical results.

The goodness-of-fit metrics are summarised in Table 5. Quantitatively, T(t) shows high agreement with the experimental measurements, with an $R^{2}$ of 0.9433 and an RMSE of 2.4887, which confirms the correctness of the predicted model. Meanwhile, E(t) exhibits moderate goodness-of-fit values, which can be attributed to the rapid early-stage decay of E(t) combined with a fixed time-step sampling scheme, resulting in a reduced effective statistical representation of the most significant modulus variations.

For the temperature evolution, the strong agreement with Newton’s law of cooling indicates that convective heat transfer governs the cooling process, resulting in faster solidification than that typically observed in conventional printing conditions. Although uncertainties in absolute temperature measurements may affect the quantitative magnitude of both T(t) and the derived E(t), the relative evolution trends remain consistent. Hence, the mechanistic interpretation and instability analysis based on these trends are not significantly influenced. The results of the parameters also stand for they are fitting with Newton’s cooling law equation as follows:

(22) $$T\left(t\right)=\left(T_{0}-T_{\infty }\right)e^{-kt}+T_{env}$$

where the cooling constant $k=\frac{hA}{mc_{p}}$.

Compared with previous studies that primarily modelled thermal evolution or sagging behaviour under specific extrusion and cooling conditions [12,21], the present results further indicate that the rapid cooling-induced stiffness evolution of unsupported strands provides the thermo-mechanical basis for subsequent structural instability in support-free bridging.

### 3.2. Strand Curvature Analysis

**Figure 15:**
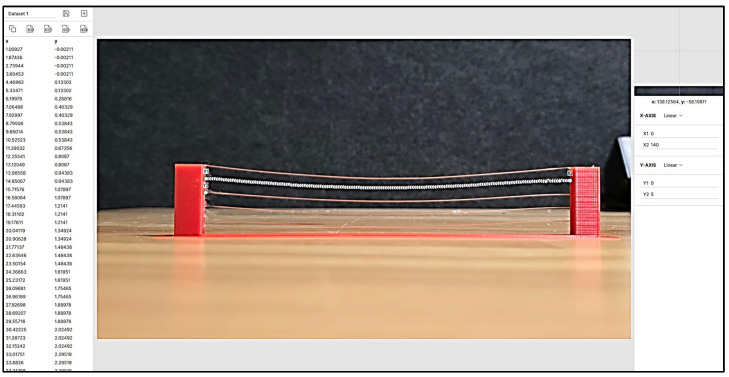
Extraction of the curvature data with PlotDigitizer^®^ Pro v3.

The curvature data of the single-strand bridging was extracted using PlotDigtizer^®^ Pro v3 (see Figure 15) from captured images taken with digital cameras. The software can provide higher accuracy based on the image resolution compared to measuring with instruments such as a vernier calliper (0.1 mm). Uncertainties in the image data affect the accuracy of the extracted values, potentially due to the camera’s perspective and distance from the object.

**Figure 16:**
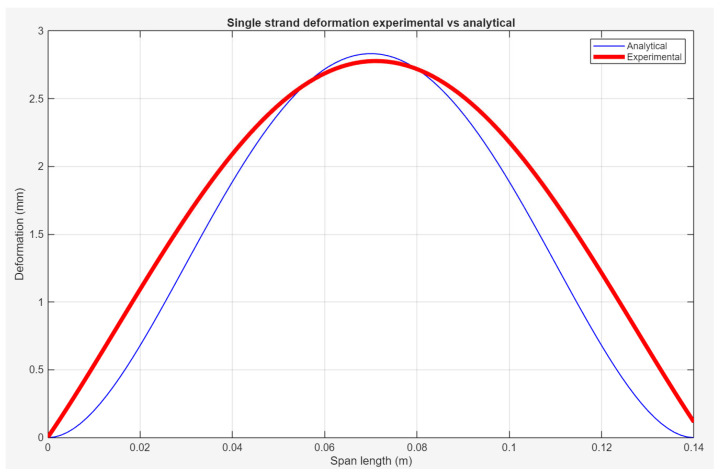
Comparison of the deflections between analytical and experimental studies.

Figure 16 presents a comparison of the single-strand bridging deflections between analytical modelling and experimental data. The goodness-of-fit results in Table 6 indicate strong agreement between the experimental data and the fitted curve, with a high coefficient of determination ($R^{2}=\;0.8322$) and a low RMSE of 0.3961.

In addition, the analytical model curve qualitatively agrees well with the fitting curve, especially in the high similarity of the peak deflection within the same span. However, discrepancies are observed near the boundary regions at both ends of the strand. The analytical model shows a more gradual variation compared to the fitting curve, which is closer to the observation in Figure 16. This discrepancy is attributed to simplified boundary assumptions and idealised material behaviour adopted in the analytical model, which do not fully capture local effects at the strand ends.

The overall agreement between the analytical model and the experimental results suggests that elongation effects during the viscoelastic stage have a negligible influence on the final bridging deformation for a single strand. Furthermore, the maximum deflection identified along the span represents the minimum geometric tolerance expected in the printed results. These findings provide a foundation for the subsequent structural and buckling analyses.

In contrast to existing studies that focus on local filament sagging or data-driven prediction of strand deformation [21,22], the present curvature analysis serves as a transitional link between single-strand behaviour and the collective deformation of multi-strand bridging structures.

### 3.3. Buckling’s Critical Condition and Mitigation

*Critical print speed during the printing process*

**Figure 17:**
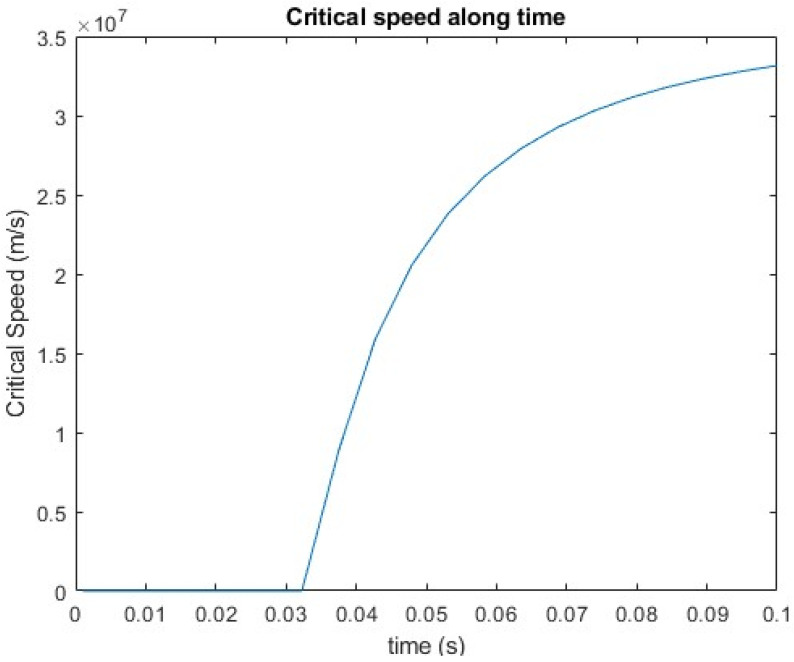
Critical print speed of buckling over time.

The critical condition of the printing process is constantly changing due to the temperature-dependent elastic modulus and other material properties. The power law equation was employed to identify the critical parameters during the printing process. The critical print speed that may lead to buckling is shown in Figure 17. In this case, the set time interval was Δt=0.01 s and the minimum was 0.27 m/s, with a rapid increase observed afterwards. The minimum critical speed increased with the increased Δt, indicating that buckling is unlikely to occur for most printing processes.

*Critical buckling conditions*

**Figure 18:**
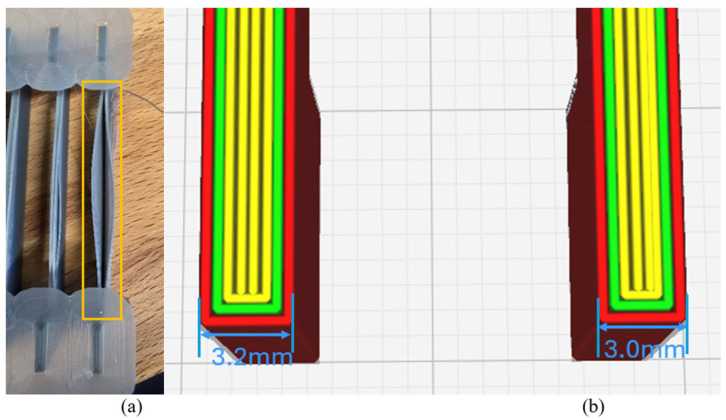
(**a**) Cross-sectional view of sliced girder component (as shown in yellow box) (**b**) delamination due to improper slicing.

According to Section 2.3.2, the key parameters of horizontal buckling can be identified, which include the span length $L_{0}$, and nozzle temperature $T_{n}$. Several test samples with various dimensions are first printed with the default print settings to check that the critical conditions are working. According to the calculations, the contribution of gravity is found to be relatively small under the studied conditions. Instead, the trigger condition is affected by three main factors: girder section dimensions, print length, and temperature difference. However, the buckling in a transverse direction is more affected by delamination; therefore, horizontal buckling is being observed for the buckling study. The critical buckling dimension was obtained by printing multiple test samples with incremental widths at the same interval depicted in Figure 18. Thus, the gradual buckling mitigation can be observed, and the critical dimension is determined with a more precise interval of width.

In practice, the girder width was defined as an integer multiple of the printing profile width to ensure consistent slicing and strand contact and to ensure the strands were in close contact rather than being loosely spaced due to slicing software limitations. Such unintended spacing may lead to inaccurate evaluation of the critical buckling condition and the delamination in the transverse direction, as shown in Figure 18a. For instance, in Figure 18b, the following test samples have girder widths of 3.2 mm (left) and 3.0 mm (right), respectively. It was observed that both were sliced using the same number of strands, but the 3.0 mm width sample exhibited reduced spacing between strands.

Critical width W refers to the equivalent threshold girder width at which buckling is first observed, determined from a series of discrete test samples with widths defined as integer multiples of the printing profile width. Table 7 shows the results of a parametric study on critical conditions of horizontal buckling that was conducted to find out the effect of the related parameters addressed in previous analytical modelling. Seven groups of testing were conducted; two FFF printers and various nozzle temperatures, span lengths, print speeds, and infill patterns were applied for the study. Additionally, each sample was repeated three times to ensure the accuracy of the results.

**Figure 19:**
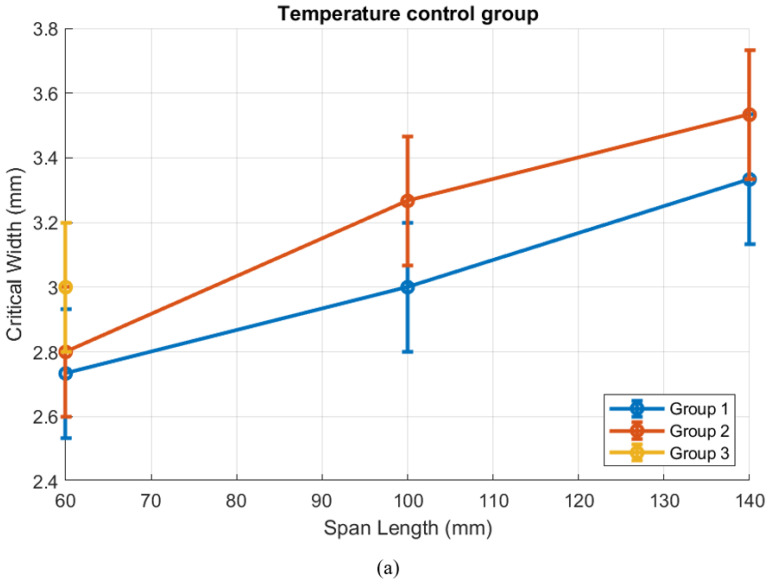

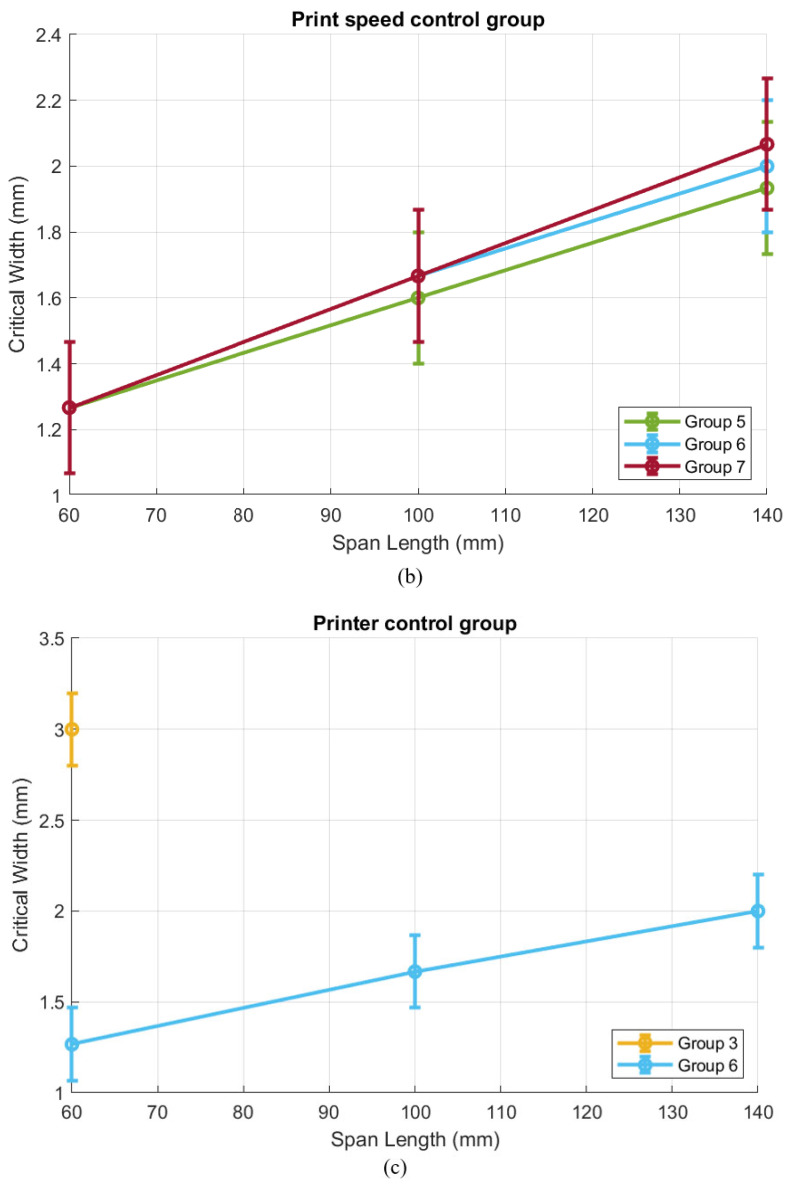

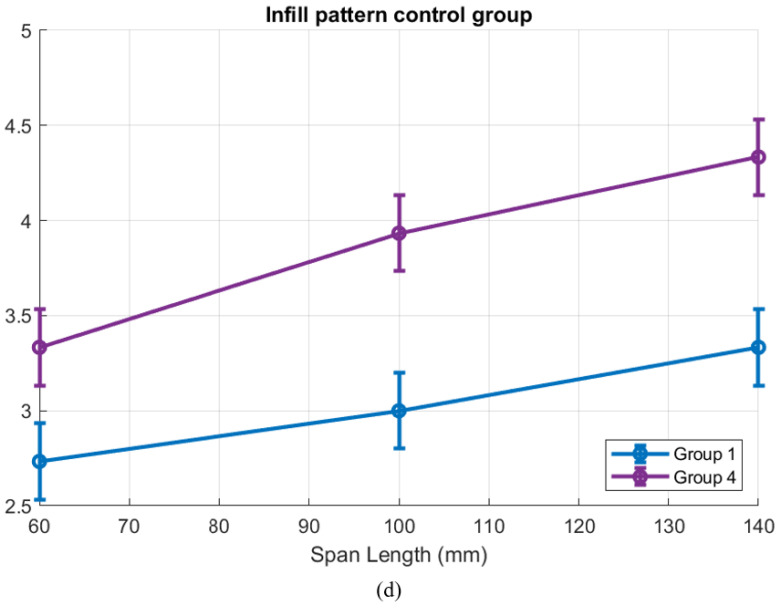
Group study of the horizontal buckling critical condition: (**a**) temperature control group, (**b**) print speed control group, (**c**) printer control group, and (**d**) infill pattern control group.

The data groups are compared to identify the difference in critical width caused by those related factors, as shown in Figure 19. The deviation due to the limitation of printing profile accuracy is considered in the following discussion.

The experiment results reveal a loose linearity between the critical width W and the span length $L_{0}$, as anticipated by the analytical modelling. This trend supports the validity of the model and provides a valuable basis for design guidance. The critical width W also changes accordingly while related factors change, specifically $T_{n}$. Meanwhile, certain factors indirectly involved in the analytical equations, such as infill patterns, may also influence the critical conditions. Quantification is difficult due to its non-numerical nature. Print speed shows limited influence on the critical width itself, but it affects the extent of buckling, which tends to result in larger deformations. Considering that non-numerical factors can also affect the solutions of critical conditions, $M_{p}$ can be considered more as a qualitative measure of buckling severity than as an exact physical value. However, $M_{p}$ still provides insights into the way of stabilising structure integrity and thus reducing the severity of buckling critical conditions.

The printer performance difference also contributes to the variability. Printer 1 failed to replicate results obtained with the same settings as Printer 2, which indicates that printing machine stability and hardware characteristics play a crucial role in determining critical conditions.

Compared with existing analytical and experimental studies on buckling in extrusion-based additive manufacturing [29,36], the present results provide quantified geometric ranges and process-related factors influencing buckling behaviour in support-free, multi-strand bridging beams, while the proposed analytical solutions offer mechanistic insight into the important effects of these factors.

It can be concluded that the buckling critical condition pattern can be considered as a linear relationship and can be used to guide the design of the geometry. The indicator $M_{P}$ represents the severity of the triggered buckling critical condition. Therefore, several strategies are recommended to reduce buckling occurrence as follows:

- Optimised geometric design. The span length and girder cross-sectional dimension should be carefully designed to prevent buckling in critical directions. Enhance structural stability by increasing the contact area between the pier and the build plate.
- FFF printing settings. Increase the nozzle temperature and reduce the print speed to improve interlayer adhesion, thereby decreasing the chance of delamination.

### 3.4. Amendments for Design for Additive Manufacturing (DfAM) of PLA

Bridging behaviours in FFF-printed PLA beams are a result of the complex interaction of multiple factors, which cannot be controlled solely by limiting the span length. The bridging defects are not only a result of bad CAD geometry but are also strongly affected by the thermal-dependent filament properties, the structural configuration of the FFF-printed part, and slicing settings. Based on the obtained results in this study, the following amendments to the DfAM of PLA can be given:

- The effect of other geometrical dimensions, in addition to the span length, is also demonstrated in this study. The current Design for Additive Manufacturing (DfAM) guidelines can be refined, especially on the common rules of thumb. Traditional DfAM methods usually rely on a fixed limit for bridging, such as the span length. Moreover, the effect of materials’ thermal and mechanical properties is neglected.
- A more comprehensive strategy can be devised from the experimental and analytical results for choosing the geometric dimensional range to effectively prevent buckling. These criteria can be incorporated into the DfAM principles to enhance structural reliability in PLA-based FFF printing processes. Moreover, the study broadens the understanding of bridging limitations by integrating dynamic thermo-mechanical considerations, which helps extend the applicability of DfAM to more complex geometries and loading conditions.
- In practice, designers can use the developed strategy to inform geometry decisions, specifically regarding buckling of the bridging defects. Therefore, the print success rate can be improved with less trial and error in CAD development, making a more efficient workflow in DfAM.

## 4. Conclusions

This study indicates buckling as a critical bridging defect that can compromise structural integrity for polymer-based FFF technology. A set of analytical models is developed and validated with the experimental studies, and the following conclusions are obtained:

- The local printing process is investigated to understand the force distribution and temperature evolution in the support-free extruded filament. The strand curvature during and after the printing process is analysed as the beam deflection and validated by the image-based measurement, with a good agreement between the experimental and analytical results, with high coefficients of determination, $R^{2},\;of\;up\;to\;0.94$.
- High cooling rates caused by the lack of direct contact with the build plate are identified as the major cause of weak interlayer adhesion.
- Structural deformation is observed in both transverse and horizontal directions, indicating the influence of residual thermal stress.
- A set of analytical models is employed to explain the buckling behaviour and is validated by parametric studies over key influencing factors.
- Thermal evolution, time-dependent material stiffness, and buckling instability are linked to bridging defects in FFF.
- Critical buckling conditions are experimentally determined with critical girder width increases from around 1.2 mm to 4.3 mm as the span length increases from 60 mm to 140 mm.
- Buckling can be controlled by optimising CAD design dimensions and adjusting print settings according to the safe range of span lengths and widths.

Future work will focus on factors that were not fully addressed in this study, such as material-specific thermal properties, e.g., coefficient of thermal expansion (CTE) and elastic modulus, and their effects on buckling behaviour. A non-linear Finite Element Analysis (FEA), including temperature-dependent and spatially non-homogeneous material behaviour, will be conducted to provide a more comprehensive assessment of the global buckling behaviour rather than focusing solely on the buckling event. Moreover, the influence of slicing strategies, such as infill patterns, on the development of buckling remains an open question and warrants further investigation. The study of filament interlayer adhesion should be integrated with the current work, as both are key contributors to delamination, which is an essential factor in bridging defects. The adhesion study can further quantify the bending moment indicator $M_{p}$. Furthermore, DfAM design guidance should be adapted to the specific material properties, structural features, and environmental influences.

## Figures and Tables

**Table 1:** Settings of FFF printing parameters.

Print Settings	Value
Print speed (mm/s)	45
Ambient temperature (°C)	25
Bed temperature (°C)	65
Infill pattern	Linear
Nozzle temperature (°C)	215
Layer height (mm)	0.2

**Table 2:** Key geometric dimensions of test samples.

Test Sample	Key Dimensions	Unit (mm)
Single-girder bridging beam structure	*L*	60 100 140
	*W*	0.2 0.4 0.6 0.8 1 2 5 10 15 20 25
	*H*	0.2 0.4 0.6 0.8 1 2 5 10 15 20 25
Multi-girder bridging beam structure	*L*	60 100 140
	*W*	0.2 0.4 0.6 0.8 1 2 5 10
	*H*	0.2 0.4 0.6 0.8 1 2 5 10

**Table 3:** Thermal modelling parameters.

Parameters	Symbol	Value	Description/Source
Convective heat Transfer coefficient	*h*	10 $W\cdot m^{-2}K^{-1}$	Natural convection of air
Heat capacity	$c_{p}$	Thermal dependent	[41]
Density	ρ	1240 $kg\cdot m^{-3}$	[41]
Thermal conductivity	k	0.13 $W\cdot m^{-1}K^{-1}$	[42]

**Table 4:** Constants for fitting curves.

Constants	Values for T(t)	Values for E(t)
a	114.835	$-7.71\times 10^{6}$
b	−5.5	−1.2102
c	309.745	$2.03\times 10^{9}$

**Table 5:** Goodness-of-fit metrics for T(t) and E(t).

Metrics	T(t)	E(t)
$R^{2}$	0.9433	0.5417
Adjusted $R^{2}$	0.9428	0.5392
RMSE	2.4887	$7.74\times 10^{7}$

**Table 6:** Goodness-of-fit metrics for curvatures.

Metrics	*T(t)*	*E(t)*
$R^{2}$	0.9433	0.5417
Adjusted $R^{2}$	0.9428	0.5392
RMSE	2.4887	$7.74\times 10^{7}$

**Table 7:** Results of critical width and $M_{p}$ at the horizontal buckling critical condition.

Test Group	Printer No.	$\mathrm{Nozzle}\;\mathrm{Temperature}\;T_{n}$ (°C)	$\mathrm{Span}\;\mathrm{Length}\;L_{0}$ (mm)	$\mathrm{Print}\;\mathrm{Speed}\;v_{n}$ (mm/s)	Infill Pattern	Critical Width W (mm)	$M_{p}$
1	1	215	140	45	Linear	3.33	1.907
	1	215	100	45	Linear	3.00	1.546
	1	215	60	45	Linear	2.73	0.862
2	1	205	140	45	Linear	3.53	1.855
	1	205	100	45	Linear	3.27	1.476
	1	205	60	45	Linear	2.80	0.735
3	1	195	140	45	Linear	N.A.	N.A.
	1	195	100	45	Linear	N.A.	N.A.
	1	195	60	45	Linear	3.00	0.518
4	1	215	140	45	Triangle	4.33	2.191
	1	215	100	45	Triangle	3.93	1.594
	1	215	60	45	Triangle	3.33	0.477
5	2	195	140	25	Linear	1.93	1.094
	2	195	100	25	Linear	1.53	0.856
	2	195	60	25	Linear	1.20	0.636
6	2	195	140	45	Linear	2.00	1.127
	2	195	100	45	Linear	1.67	0.918
	2	195	60	45	Linear	1.33	0.686
7	2	195	140	65	Linear	2.20	1.224
	2	195	100	65	Linear	1.80	0.978
	2	195	60	65	Linear	1.27	0.662

## Data Availability

The datasets generated and/or analysed during the current study are not publicly available but may be made available by the corresponding author upon reasonable request, subject to approval by the journal.
